# Supplementary material for: Breeding practices and trait preferences of smallholder farmers for indigenous sheep in the northwest highlands of Ethiopia: Inputs to design a breeding program
Source: PLoS One. 2020 May 12;15(5):e0233040. doi: 10.1371/journal.pone.0233040 (PMC7217445; doi:10.1371/journal.pone.0233040)
Supplement: S4 File — The questionnaire developed to collected socioeconomic and sheep breeding practice data. (PDF) [file pone.0233040.s004.PDF]

Date ________________________________

Questionnaire

The aim is to collect data on smallholder farmers' sheep breeding practices that can be used to design a breeding program for sustainable use of sheep genetic resources in the northwest highlands of Ethiopia.

Do you agree to provide the information for the purpose described above? Yes/no, if yes continue or stop otherwise.

I. General information

1. Respondent's code _________________ districts ________________ kebele______
2. Respondent's sex: 1) male 2) female
3. Educational status of the respondent:

   1) Illiterate 4) Elementary school

   2) Writing and reading 5) Secondary school

   3)Spiritual/religious 6) Above secondary school

4. What is your main occupation? 1) crop production 2) livestock production 3) both crop and livestock production 4) trading 5) other (specify)__________________________________
5. Do you have crop land? yes/no, if yes, how many hectare? _______
6. Do you have private grazing land? Yes/no, if yes, how many hectare? ___________
7. Do you have access for grazing land? Yes/no, if yes, in which season of the year do you graze sheep? ____

II. Sheep population and purpose of rearing

8. Please tell us the number of sheep currently available in your sheep flock based on the table below.

Category	Number
Number of the breeding ewe(s)	
Number of breeding ram(s)	
Number of ewe lambs between 6 months and 1 year age	
Number of ram lambs between 6 months and 1 year age	
Number of male and female lambs below 6 months of age	
Number of castrated male sheep	

9. Please rank the four major objectives of sheep production among those listed in the table below.

Purposes	Rank
Income generation	
Meat source for conception	
Wool/hair production	
Means of saving money	
Manure production (fertilizer, fuel source, etc.)	
Skin for various purpose including selling	
Others (specify)	

III. Sheep herding and management practice

10. How is sheep flock herded during the day time especially with respect to age and sex category? 1) Males and females are separated 2) Lambs are separated 3) All classes of sheep are herded together 4) other (specify) ____

11. How is the way of sheep rearing? 1) Sheep of a household run as a flock year round 2) Sheep of more than one household run as a flock year round 3) flocks of more than one household mixed during non-cropping season 4) other (specify)___________________________

12. Do you have the experience of using sheep manure for different purposes? Yes/no.

    If yes, mention the purposes____

13. Do you shear sheep for wool/fleece production? Yes/no.

    13.1.If yes, how often you practiced? ____

    13.2.For what purposes do you use the wool/fleece? ________________________________

14. Which of the following management activities and how often do you perform?

Activities	For which sheep group	How often per year
Deworming internal parasite		
Spraying for external parasite		
Dehorning		
Hoof trimming		
Vaccination		

IV. Reproduction characteristics of ewes

15. What is the average reproductive life time of ewes? ______________________years

16. What is the average number of lambing per ewe lifetime? ________________years

V. Ram use and mating practices

17. Do you have own ram? Yes/no.
    - 17.1 If yes, how many rams do you have? _____
    - 17.2 If more than one, why do you need to keep more than one ram? ___________
    - 17.3 Where is the source of ram? 1) purchased privately 2) purchased with partner 3) born in the flock 4) other (specify) ____
18. For how many years on the average is the same breeding ram can serve in the flock? _______
19. If you do not have own breeding ram, where is the ram source? 1) neighbors and relatives 2) Unknown ram during herding 3) Others (specify) ___________
20. Do you control mating and lambing seasons for your sheep flock? Yes/no, if yes, which seasons are preferred for mating and lambing
21. Do you practice selection for breeding rams? Yes/no. if yes, please rank four major selection criteria among those listed in the table

Criteria	Rank	Remarks
Body size		
Coat color		
Growth rate		
Adaptability		
Wool/hair		
Horns		
Age		
Libido/sexual activity /		
Tail type/length		
Ear size		
Pedigree information		
Other (specify)		

22. Do you practice selection for breeding ewes? Yes/no. if yes, please rank four major selection criteria among those listed in the table

Criteria	Rank	Remarks
Body size		
Coat color		
Growth		
Adaptability		
Wool/hair		
Horns		
Age at sexual maturity		
Lambing interval		

Tail type/length		
Twining ability		
Lamb survival		
Lamb growth		
Ear size		
Pedigree information		
Other (specify)		

23. Do you practice the culling of sheep? Yes/no. If yes, which reasons are applicable?

Reasons	Female sheep	Male sheep
Small body size		
Unwanted coat color		
Poor body condition		
Old age		
Sterility *		
Repeated abortion *		
Poor mothering ability		
Other (specify)		

*Fertility problems

24. If you practice culling of ram due to old age, what is the average culling age? __________years
25. If you practice culling of ewes due to old age, what is the average culling age? _________years
26. If culling is due to unwanted coat colors, which coat color(s) is/ are regarded as unwanted for breeding purposes in male and female sheep?____

Thank you very much!
