# Supplementary material for: Breeding practices and trait preferences of smallholder farmers for indigenous sheep in the northwest highlands of Ethiopia: Inputs to design a breeding program
Source: PLoS One. 2020 May 12;15(5):e0233040. doi: 10.1371/journal.pone.0233040 (PMC7217445; doi:10.1371/journal.pone.0233040)
Supplement: S5 File — The questionnaire translated from English into the local language (Amharic). (PDF) [file pone.0233040.s005.PDF]

ቀን __________

<u>ለአርሶ አደሮች የተዘጋጀ መጠይቅ</u>

ዓላማ፡ አ/አደሩ ያለውን የበግ አረባብ ልምድና ስርዓት እንደዚሁም የወደፊት ፍላጎቱን የሚያሳይ መረጃ በመሰብሰብና በመተንተን የአካባቢ በግ ምርታማነትን ለማሻሻል የሚረዳ እቅድ ለማቀድ የተዘጋጀ ነው።

ከላይ በተገለጸው መሰረት የሚፈለገውን መረጃ ለመስጠት ተስማምተዋል? ከተስማሙ መረጃውን ይሙሉ

I. አጠቅላይ መረጃ

1.የተጠያቂው መለያ ኮድ _______ ወረዳ_______ቀበሌ_____________

2.የተጠያቂው ጾታ፡ ወንድ/ሴት______

3.የተጠያቂው የትምህርት ደረጃ፡ 1)ያልተማረ 2)መጻፍና ማንበብ የሚችል 3) መንፈሳዊ ትምህርት የተማረ 4) 1ኛ ደረጃ ድረስ የተማረ 5) ሁለተኛ ደረጃ ድረስ የተማረ 6) ከሁለትኛ ደረጃ በላይ የተማረ

4.በዋናነት የሚሰሩት የስራ መስክ ምንድን ነው? 1) ሰብል ማምረት 2) እንስሳት ማርባት 3) ሰብልና እንስሳት ጥምር ግብርና 4) ንግድ 5) ሌላ ካለ ይገለጽ ____________

5.ምን ያህል የእርሻ መሬት አለዎት? _________ ጥማድ/ሄክታር

6. ምን ያህል የግል የግጦሽ መሬት አለዎት? _________ ጥማድ/ሄክታር፣

7.የወል የግጦሽ መሬት ይጠቀማሉ? የሚጠቀሙ ከሆነ በየትኛው ወቅት ለበጎች ይጠቀማሉ?____________

____

II. የበግ ብዛትና የእርባታ ጥቅም በተመለከተ

8. በአሁኑ ወቅት ምንያህል በግ እንዳለዎት ቢነግሩኝ

የበግ መለያ/ደረጃ	ብዛት
የእርባታ እናት በጎች ብዛት	
የእርባታ አውራ በጎች ብዛት	
እድሚያቸው ከ6 ወር እስከ አንድ አመት የሆነ የቄብ በግ ብዛት	
እድሚያቸው ከ6 ወር እስከ አንድ አመት የሆነ የወንድ በግ ወጠጤ ብዛት	
እድሜያቸው ከ6ወር በታች የሆነ ወንድና ሴት ግልገል ብዛት	
የተኮላሹ አውራ በጎች ብዛት	

9. ከሚከተሉት የበግ ማርባት ጥቀሜታዎች ከእርስዎ እይታ አንጻር በማየት አራቱን በቅደም ተከተል ቢያስቀምጡልኝ? ዋና ጥቅም ለሆነው ደረጃ 1 ሰጥተው ለቀጣዩ 2፣ 3 ና 4 እያሉ መስጠት ይችላሉ

የበግ ማርባት ጠቀሜታ	ደረጃ
ሸጦ ገንዘብ ለማግኘት	
የበግ ጸጉር አምርቶ ለመሸጥ	
ለስጋ ወይም ለእርድ አገልግሎት	
ገንዘብ መቆጠቢያ ዘዴ	
የበግ ፍግን ለተለያየ አገልግሎት ለመጠቀም	
የበግ ሌጦ (ቆዳ) ለመሸጥ	
ሌላ የተለየ ጥቅም ካለ ቢገለጽ	

III. የበግ እንክብቤን በተመለከተ

10. በግ የመጠበቅ ልምድዎ ምን ይመስላል? 1) ወንድና ሴት በግ በአንድ ላይ ይጠበቃል 2) ግልገሎች ለብቻ ይጠበቃሉ 3) ሁሉም በጎች በአንድ ላይ የጠበቃሉ

11. አብዛኛውን ጊዜ በጎች ለግጦሽ መስክ በሚሄዱበት ጊዜ የራስዎን በግ ለብቻ ነው የሚያሰማሩት ወይስ ከሌሎች ሰዎች ጋር በመቀላቀል? ________________

12. የበግ ፍግን/በጠጥ/ ለተለያዩ አገልግሎቶች የመጠቀም ልምድ አለዎት? መልስዎ አዎ ከሆነ ለምን ለምን እንደሚጠቀሙ ቢገልጹልኝ ____

13. የበጎችን ጸጉር ይሸልታሉ? __________መልስዎ አዎ ከሆነ በዓመት ስንት ጊዜ?________

ጸጉሩ ለምን አገልግሎት ይጠቀሙበታል?______________________

14. ከሚከተሉት ወስጥ የትኞቹን ለበጎችዎ እንክብክቤ ያደርጋሉ?

ዝርዝር	በየትኛው እድሜ ክልል ላሉ በጎች	በዓመት ስንት ጊዜ
የውስጥ ጥገኛ ህክምና (ኪኒን)		
የውጭ ጥገኛ ርጭት		
ቀንድ ማስቆረጥ (ያለአግባብ ያደገ)		
ጥፍር ማስቆረጥ (ያለአግባብ ያደገ)		
ክትባት ማስከተብ		

IV. የእናት በግን በተመለከተ

15. እናት በጎች ግልገል እየወለዱ በአማካኝ ለመን ያህል ዓመት ይቆያሉ? __________

16. እናት በጎች በህይወት ዘመናቸው ምን ያህል ጊዜ ይወልዳሉ? __________

V. የበግ አረባብ ዘዴን በተመለከተ

17. የራስዎ የሆነ ለእርባታ የሚሆን አውራ አለዎት?_____________

17.1 መልስዎ አዎ ከሆነ ለማጥቃት የደረሰ ሰንት አወራ በግ አለዎት?___________

17.2 ከአንድ በላይ አውራ ያለዎት ከሆነ ለምን እንደሚጠቀሙበት ቢገልጹ? ________________

17.3 የሚጠቀሙበትን አውራ ከቤትዎ የተወለደ ነው ወይስ በግልዎ የገዙት ወይስ ከሌሎች ሰዎች ጋር በጋራ የገዙት?

18. አንድን አውራ ለስንት ዓመት ይጠቀሙበታል?________

19. አውራ ከሌለዎት ሴት በጎችን የሚያስጠቁት የት ነው? 1) ከጎረቤት አውራና ከዘመድ 2) መስክ ላይ አብረው ሲውሉ በተገኘው አውራ 3) ሌላ መንገድ ካለ ቢገለጽ __________

20. የበጎችን የመጠቂያና የመውለጃ ጊዚያት ይወስናሉ? የተመረጡ የመራቢያ ወቅቶችን የሚጠቀሙ ከሆነ የትኞቹን

____

21. ለአውራ የሚሆን ወንድ በጎችን መርጠው ያረባሉ? ___________ መልስዎ አዎ ከሆነ ከሚከተሉት የአውራ በግ መምርጫ መስፈርቶች መካከል እርስዎ በዋናነት የሚጠቀሙባቸውን አራት ዋና መስፈርቶችን በቅደም ተከተል ቢያስቀምጡ

መስፈርት	ደረጃ	መስፈርት	ደረጃ
የሰውነት መጠን/አቋም		እድሜ	
መልክ		የማጥቃት ብቃት	
የእድገት ሁኔታ		የላት መጠን	
ድርቅና ረሃብ የሚቋቋም		የጆሮ መጠን	
ጸጉር		ዝርያውን በመጠየቅ	
ቅንድ በማየት		ሌላ ካል ይገለጽ	

22. እናት በጎችን መርጠው ያረባሉ? _________________ መልስዎ አዎ ከሆነ ከሚከተሉት የእናት በግ መምርጫ መስፈርቶች መካከል በዋናነት የሚጠቀሙባቸውን አራት ዋና መስፈርቶችን በቅደም ተከተል ቢያስቀምጡ

መስፈርት	ደረጃ	መስፈርት	ደረጃ
የሰውነት መጠን/አቋም		የላት መጠን	
መልክ		መንታ አወላለድ	
የእድገት ሁኔታ		የግልገል አስተዳደግን በማየት ከጤና አንጻር	
ድርቅና ረሃብ የምትቋቋም		የግልገሎች እድገት	
ጸጉር		የጆሮ ሁኔታ	
ቀንድ በማየት		ዝርያ በመጠይቅ	
ቶሎ ለመጠቃት የምትደርስ		ሌላ ካለ ቢገለጽ	
ቶሎቶሎ ግልገል የምትዎልድ			

23. ለእርባታ የማይሆኑ ውንድና ሴት በጎችን ያስውግዳሉ?______________________ መልስዎ አዎ ከሆነ የትኞቹን ምክንያቶች ይጠቀማሉ?

ምክንያት	ሴት በግ	ወንድ በግ
የሰውነት መጠን/አቋም ትንሽ በመሆኑ		
የማይፈለግ መልክ ሲሆን		
ሰውቱ/ቷ በጣም የከሳ ሲሆን		
የእድሜ እርጅና		
መውለድ/ማጥቃት አለመቻል/በተደጋጋሚ ማስወረድ/ ቶልቶል አለመውለድ		
ጥሩ ግልገል ማሳደግ የማትችል ከሆነ		
ሌላ ካለ ቢገለጽ		

24. ወንድ በግ በእርጅና ምክንያት የሚወገድ ከሆነ በአማካኝ ስንት ዓመት ሲሆነው ነው አረጇ ተብሎ የሚዎገደው? ________________________

25. ሴት በግ በእርጅና ምክንያት የምትወገድ ከሆነ በአማካኝ ስንት ዓመት ሲሆነው ነው አረጀች ተብላ የምትዎገደው? ____

26. ለወንድና ሴት በግ የማይፈለግ ምልክ የሚባልው የትኛው ቀለም ነው? ______________________________

እናመሰግናለን!
