# Supplementary material for: Breeding practices and trait preferences of smallholder farmers for indigenous sheep in the northwest highlands of Ethiopia: Inputs to design a breeding program
Source: PLoS One. 2020 May 12;15(5):e0233040. doi: 10.1371/journal.pone.0233040 (PMC7217445; doi:10.1371/journal.pone.0233040)
Supplement: Attachment — Submitted filename: PONE-D-20-02104_reviewer.pdf [file pone.0233040.s006.pdf]

PLOS ONE

Breeding practices and trait preferences of smallholder farmers for indigenous sheep in the northwest highlands of Ethiopia: Inputs to design a breeding program

--Manuscript Draft--

**Manuscript Number:**	PONE-D-20-02104
**Article Type:**	Research Article
**Full Title:**	Breeding practices and trait preferences of smallholder farmers for indigenous sheep in the northwest highlands of Ethiopia: Inputs to design a breeding program
**Short Title:**	Breeding practices and trait preferences of smallholder farmers for indigenous sheep
**Corresponding Author:**	Abiye Shenkut Abebe<br>Bahir Dar University College of Agriculture and Environmental Sciences<br>Bahir Dar, ETHIOPIA
**Keywords:**	Breeding objective; trait preference; choice experiment; community-based breeding program; Ethiopian indigenous sheep breeds; sustainable genetic improvement; choice modeling; smallholder farmers' indigenous knowledge about sheep breeding; breeding rams and ewes; sheep selection and culling criteria; coat color; body size
**Abstract:**	The aim of this study was to identify breeding practices and trait preferences for indigenous sheep in three districts (Estie, Farta and Lay Gayient) located in the northwest highlands of Ethiopia. Questionnaire survey and choice experiment methods were used to collect data from 370 smallholder farmers. Respondents were selected randomly among smallholder farmers who own sheep in the aforementioned districts. Choice modeling using a generalized multinomial logit model was employed to examine preferences for sheep attributes while descriptive statistics and index values were computed to describe sheep breeding practices. Having the highest index value of 0.36, income generation was ranked as the primary reason for keeping sheep followed by meat and manure sources. The average flock size per smallholder farmer was 10.21 sheep. The majority of the smallholder farmers (91%) have the experience of selecting breeding rams and ewes within their own flock using diverse criteria. Given the highest index value of 0.34, body size was ranked as a primary ram and ewe selection criteria followed by coat color. Furthermore, choice modeling results revealed that tail type, body size, coat color, growth rate, horn and ear size have shown significant influences on smallholder farmers' preference for breeding rams (P<0.01). The part-worth utility coefficients were positive for all ram attributes except ear size. For breeding ewes, mothering ability, coat color, body size, lambing interval, growth rate, tail type and litter size have shown significant effects on choice preferences of smallholder farmers (P<0.05). Moreover, significant scale heterogeneity was observed among respondents for ewe attributes (P<0.001). Overall, the results implied that sheep breeding objective suitable for the northwest highlands of the country can be derived from traits such as linear body measurement, weight and survival at different ages, and lambing intervals. However, selection decisions at the smallholder level should not only be based on estimated breeding values of traits included in the breeding objective but instead, incorporate ways to address farmers' preference for qualitative traits.<br>Keywords: breeding objective, choice experiment, indigenous sheep, selection criteria
**Order of Authors:**	Abiye Shenkut Abebe
	Kefyalew Alemayehu
	Anna Maria Johansson
	Solomon Gizaw
**Additional Information:**	
**Question**	**Response**
**Financial Disclosure**<br><br>Enter a financial disclosure statement that	ASA received fund for this study from the Ethiopian Ministry of Education via College of Agriculture and Environmental Sciences of Bahir Dar University (Ref.No/250/2019). The website for College of Agriculture and Environmental Sciences of Bahir Dar

Powered by Editorial Manager® and ProduXion Manager® from Aries Systems Corporation

describes the sources of funding for the work included in this submission. Review the submission guidelines for detailed requirements. View published research articles from *PLOS ONE* for specific examples.<br><br>This statement is required for submission and **will appear in the published article** if the submission is accepted. Please make sure it is accurate.<br><br>**Unfunded studies**<br>Enter: *The author(s) received no specific funding for this work.*<br><br>**Funded studies**<br>Enter a statement with the following details:<br>• Initials of the authors who received each award<br>• Grant numbers awarded to each author<br>• The full name of each funder<br>• URL of each funder website<br>• Did the sponsors or funders play any role in the study design, data collection and analysis, decision to publish, or preparation of the manuscript?<br>• **NO** - Include this sentence at the end of your statement: *The funders had no role in study design, data collection and analysis, decision to publish, or preparation of the manuscript.*<br>• **YES** - Specify the role(s) played.<br><br>* typeset	University is https://bdu.edu.et/caes/. The funders had no role in study design, data collection and analysis, decision to publish, or preparation of the manuscript.
**Competing Interests**<br><br>Use the instructions below to enter a competing interest statement for this submission. On behalf of all authors, disclose any competing interests that could be perceived to bias this work—acknowledging all financial support and any other relevant financial or non-financial competing interests.<br><br>This statement **will appear in the published article** if the submission is accepted. Please make sure it is accurate. View published research articles from *PLOS ONE* for specific examples.	The authors have declared that no competing interests exist

Powered by Editorial Manager® and ProduXion Manager® from Aries Systems Corporation

**NO authors have competing interests**<br><br>Enter: *The authors have declared that no competing interests exist.*<br><br>**Authors with competing interests**<br><br>Enter competing interest details beginning with this statement:<br><br>*I have read the journal's policy and the authors of this manuscript have the following competing interests: [insert competing interests here]*<br><br>* typeset	
**Ethics Statement**<br><br>Enter an ethics statement for this submission. This statement is required if the study involved:<br><br>• Human participants<br>• Human specimens or tissue<br>• Vertebrate animals or cephalopods<br>• Vertebrate embryos or tissues<br>• Field research<br><br>Write "N/A" if the submission does not require an ethics statement.<br><br>General guidance is provided below. Consult the submission guidelines for detailed instructions. **Make sure that all information entered here is included in the Methods section of the manuscript.**	Prior to the study, data collection formats and procedures were reviewed and approved by research ethics review committee of Debre Tabor University, Ethiopia (number DTU13/19).<br>Respondents also provided their verbal informed consent to take part in this study. Furthermore, the data were analyzed anonymously and name, ethnicity and religious issues were not asked and recorded during data collection.

Powered by Editorial Manager® and ProduXion Manager® from Aries Systems Corporation

**Format for specific study types**<br><br>**Human Subject Research (involving human participants and/or tissue)**<br>• Give the name of the institutional review board or ethics committee that approved the study<br>• Include the approval number and/or a statement indicating approval of this research<br>• Indicate the form of consent obtained (written/oral) or the reason that consent was not obtained (e.g. the data were analyzed anonymously)<br><br>**Animal Research (involving vertebrate animals, embryos or tissues)**<br>• Provide the name of the Institutional Animal Care and Use Committee (IACUC) or other relevant ethics board that reviewed the study protocol, and indicate whether they approved this research or granted a formal waiver of ethical approval<br>• Include an approval number if one was obtained<br>• If the study involved *non-human primates*, add *additional details* about animal welfare and steps taken to ameliorate suffering<br>• If anesthesia, euthanasia, or any kind of animal sacrifice is part of the study, include briefly which substances and/or methods were applied<br><br>**Field Research**<br><br>Include the following details if this study involves the collection of plant, animal, or other materials from a natural setting:<br>• Field permit number<br>• Name of the institution or relevant body that granted permission	
**Data Availability**<br><br>Authors are required to make all data underlying the findings described fully available, without restriction, and from the time of publication. PLOS allows rare exceptions to address legal and ethical concerns. See the PLOS Data Policy and FAQ for detailed information.	Yes - all data are fully available without restriction

A Data Availability Statement describing where the data can be found is required at submission. Your answers to this question constitute the Data Availability Statement and **will be published in the article**, if accepted.<br><br>**Important:** Stating 'data available on request from the author' is not sufficient. If your data are only available upon request, select 'No' for the first question and explain your exceptional situation in the text box.<br><br>Do the authors confirm that all data underlying the findings described in their manuscript are fully available without restriction?	
**Describe where the data may be found in full sentences. If you are copying our sample text, replace any instances of XXX with the appropriate details.**<br><br>• If the data are **held or will be held in a public repository**, include URLs, accession numbers or DOIs. If this information will only be available after acceptance, indicate this by ticking the box below. For example: *All XXX files are available from the XXX database (accession number(s) XXX, XXX.).*<br>• If the data are all contained **within the manuscript and/or Supporting Information files**, enter the following: *All relevant data are within the manuscript and its Supporting Information files.*<br>• If neither of these applies but you are able to provide **details of access elsewhere**, with or without limitations, please do so. For example:<br><br>*Data cannot be shared publicly because of [XXX]. Data are available from the XXX Institutional Data Access / Ethics Committee (contact via XXX) for researchers who meet the criteria for access to confidential data.*<br><br>*The data underlying the results presented in the study are available from (include the name of the third party*	All relevant data are within the manuscript and its Supporting Information files

and contact information or URL).<br>• This text is appropriate if the data are owned by a third party and authors do not have permission to share the data.<br><br>* typeset	
Additional data availability information:	

Powered by Editorial Manager® and ProduXion Manager® from Aries Systems Corporation

Manuscript Click here to download Manuscript manuscript.docx

Breeding practices and trait preferences of smallholder farmers for indigenous sheep in the northwest highlands of Ethiopia: Inputs to design a breeding program

Abiye Shenkut Abebe[1, 2*], Kefyalew Alemayehu[1], Anna M. Johansson[3], Solomon Gizaw[4]

[1] Department of Animal Production and Technology, Bahir Dar University, Bahir Dar, Ethiopia

[2] Department of Animal Science, Debre Tabor University, Debre Tabor, Ethiopia

[3] Department of Animal Breeding and Genetics, Swedish University of Agricultural Sciences, Uppsala, Sweden

[4] International Livestock Research Institute (ILRI), Addis Ababa, Ethiopia

[*] Corresponding author

Email: abiysh85@gmail.com

// Abstract

The aim of this study was to identify breeding practices and trait preferences for indigenous sheep in three districts (Estie, Farta and Lay Gayient) located in the northwest highlands of Ethiopia. Questionnaire survey and choice experiment methods were used to collect data from 370 smallholder farmers. Respondents were selected randomly among smallholder farmers who own sheep in the aforementioned districts. Choice modeling using a generalized multinomial logit model was employed to examine preferences for sheep attributes while descriptive statistics and index values were computed to describe sheep breeding practices. Having the highest index value of 0.36, income generation was ranked as the primary reason for keeping sheep followed by meat and manure sources. The average flock size per smallholder farmer was 10.21 sheep. The majority of the smallholder farmers (91%) have the experience of selecting breeding rams and ewes within their own flock using diverse criteria. Given the highest index value of 0.34, body size was ranked as a primary ram and ewe selection criteria followed by coat color. Furthermore, choice modeling results revealed that tail type, body size, coat color, growth rate, horn and ear size have shown significant influences on smallholder farmers' preference for breeding rams ($P<0.01$). The part-worth utility coefficients were positive for all ram attributes except ear size. For breeding ewes, mothering ability, coat color, body size, lambing interval, growth rate, tail type and litter size have shown significant effects on choice preferences of smallholder farmers ($P<0.05$). Moreover, significant scale heterogeneity was observed among respondents for ewe attributes ($P<0.001$). Overall, the results implied that sheep breeding objective suitable for the northwest highlands of the country can be derived from traits such as linear body measurement, weight and survival at different ages, and lambing intervals. However, selection decisions at the smallholder level should

not only be based on estimated breeding values of traits included in the breeding objective but instead, incorporate ways to address farmers' preference for qualitative traits.

Keywords: breeding objective, choice experiment, indigenous sheep, selection criteria

Introduction

Ethiopia is one of the few African countries endowed with huge sheep genetic resources. The estimated population size is about 31.3 million sheep, of which 99.8% are indigenous types [1]. Ecologically, sheep are found in diverse production environments that range from arid lowlands to extremely cool highlands. The northwest highlands are among the major sheep production areas of the country where Farta sheep breed and their crosses with other indigenous sheep are widely distributed [2, 3]. For the smallholder farmers, sheep provide valuable contributions through income generation, direct food sources, non-food utilities and various sociocultural privileges [3, 4, 5]. Particularly when crop farming is less reliable due to drought or other factors, sheep are commonly used as a means of risk mitigation and often considered as living banks for the smallholders.

Despite large abundances and diverse functions, the average productivity of indigenous sheep is generally low. For example, Mekuriaw et al. [6] reported an average yearling weight of about 20 kg for Farta sheep while Gulilat et al. [7] obtained an average carcass yield of 10 kg for the same breed. It has been known that indigenous sheep are not selected systematically for a specific purpose but instead, developed through natural selection and raised for millennia under the traditional management system. Through such episodes, indigenous sheep are assumed to be merited largely with adaptive traits that are useful to survive and reproduce in low-input and stressful production environments [8]. The problem is that the traditional breeding system

implemented at the smallholder level has not been supported with proven scientific methodologies and the state of art technologies in animal breeding. As a result, indigenous sheep have been performing poorly year in and year out, which is a common scenario in the northwest highlands.

Given the suitability of the area and adaptive potentials of the existing sheep, ~~a recent initiative has been taken to use~~ such resources can be a pathway to put smallholders ~~as a pathway~~ out of poverty in the northwest highlands of Ethiopia. However, to design and implement an effective breeding program, breeding objective traits specific to sheep breeds reared in the target areas have not yet identified. In other words, a thorough analysis of sheep breeding experiences and trait preferences is required to sensibly define breeding objectives and design genetic improvement programs at smallholder level [4, 5].

Various methodological approaches are available to identify breeding objective traits in livestock. For instance, choice experiments [9-12], ranking among a list of traits [13, 14], and live animal rankings [15]. Recently, a choice experiment has been widely applied to investigate farmers' preferences for animal traits. It provides a hypothetical depiction of attributes levels giving adequate options for the respondents to reflect their interests. Although it requires higher cognitive efforts, a choice experiment is useful to identify preferences when the number of sheep per household is very small [16]. Overall, applying a combination of methods has been suggested to effectively identify breeding objective traits [17]. As such, this study aimed at identifying breeding practices and smallholder farmers' trait preferences for indigenous sheep in the northwest highlands of Ethiopia using ranking of traits and choice experiment methods.

Materials and methods

Description of the study areas

The study was conducted in Estie, Farta and Lay Gayient districts of South Gondar Zone of the Amhara Region, located in the northwest highland of Ethiopia (Fig.1). These districts were

selected because they have huge potentials for sheep production. Furthermore, a preliminary assessment prior to the present study has identified the three districts as niche areas for Farta sheep breed, in which designing and implementing a breeding program is under consideration. The agricultural practice in these areas is crop-livestock mixed farming where livestock play invaluable roles for crop cultivation and livelihood of smallholder farmers [3]. The average annual rainfall of Estie, Farta and Lay Gayient districts is 1591, 1122 and 1200 mm, respectively. Estie and Lay Gayient districts have received similar minimum (9ºC) and maximum (22 ºC) average daily temperatures. Whereas, the minimum and maximum average daily temperatures of Farta districts are 8ºC and 18ºC, respectively.

Fig 1. Map of the study areas in South Gondar Zone of the Amhara Region.

Study approach

The study followed two approaches to acquire the data. First, data about socioeconomic status and sheep breeding experience of the respondents were collected through in-person interviews using a semi-structured questionnaire. The sheep breeding practice data includes production objectives, flock size and composition, selection and culling criteria, ram use and mating system. Second, sheep trait preferences were collected by means of a discrete choice experiment where the respondents have chosen their preferred alternative from choice sets built through hypothetical trait levels combinations.

Choice experiment design

Identifying attributes and their levels are the principal steps to design choice experiments [18]. In this study, sheep traits were identified and prioritized with focus-group discussion involving well-experienced smallholder farmers, researchers, and livestock experts. Following the discussion, six

traits for rams and seven traits for ewes were selected. Attributes with their descriptions and levels are shown in Table 1. Levels were effect-coded to minimize confounding between parameter estimates [18]. A JMP software version 14 [19] was used to construct a full factorial design containing 64 and 128 profiles for rams and ewes, respectively. However, making a choice among the full factorial could be cognitively complex for the smallholder farmers. To reduce the size of the design, a fractional factorial with resolution IV design was applied [20]. Subsequently, an orthogonal array with 16 profiles grouped into four blocks was generated. Furthermore, to prevent forced choices, an opt-out option was assigned in each block, making the number of choice sets five per block.

Table 1. Ram and ewe attributes and levels included in the choice experiment

**Attributes**	**Attribute descriptions**	**Levels with effect-coded**
**Body size**	the physical appearances including the height and body length of rams and ewes	Large = 1, small = -1
**Coat color**	the type of color predominantly observed on the body of rams and ewes	brown = 1, white = -1
**Growth**	yearling live weight in which rams and ewes reach at breeding age	Rapid = yearling weight 30kg = 1, slow = yearling weight 20kg = -1
**Lambing intervals ***	the average lambing interval between two successive lambing of ewes	short = 3 lambing in 2 years = 1, long = 1 lambing per year = -1
**Mothering ability ***	the ability of ewes to nourish their lambs that could also be implicated on lamb growth and survival	Good =1, poor = -1
**Litter size ***	the number of lambs born per ewe per lambing	twin = 1, single =-1
**Ear size $**	the size of the ears of rams	Large =1, small =-1
**Horn$**	the presence and absence of horn in rams	Horned =1, polled =-1
**Tail type**	the length and width of the tail in rams and ewes	length covering the testicular area with sufficient width for rams and extended halfway to the udder for ewes = good = 1, small and thin = bad = -1

**appeared only in ewes, $ appeared only in rams, unmarked attributes appeared in both ewes and rams*

Choice cards with ~~pictorial~~ figure representation of the different profiles of rams and ewes were prepared. Well educated enumer[illegible]s were selected and trained to collect data under the close supervision of the researchers. Prior to the collection of the actual data, the questionnaire and choice cards were pretested in the study sites. Based on the feedback, modifications were made on the questionnaire and illustrations of the profiles. A sample of final choice cards used for ewe attributes is shown in Fig 2.

Fig 2. Sample choice card for ewe attribute levels.

Sample size

To determine the minimum number of respondents required for the choice experiment, we used the method suggested by Johnson and Orme [21] and Orme [22] as:

$$N > (500 * c) / (t * a) \quad (1)$$

Where N is the sample size, c is the highest number of levels of any attribute for main effect design, t represents the number of choice tasks, a is the size of choice set per task.

In this study, the number of choice tasks per block was one with four alternatives excluding the opt-out option and a maximum of two levels per attribute. Plugging in the information to the formula resulted in a minimum sample size of 250. However, we collected the data from a total of 385 respondents randomly selected among smallholder farmers who own sheep.

Data collection

Procedurally, the respondents were asked first about their socioeconomic characteristics and experiences about sheep breeding in a face-to-face interview. Subsequently, choice cards were introduced. Following a brief explanation about the choice cards, respondents were asked to choose the most preferred hypothetical sheep for breeding purposes. The field data were collected

from March to June 2019. From the total respondents, 370 farmers (Estie = 117, Farta = 125 and Lay Gayient =128) have successfully completed both the questionnaire and the choice experiments. Only 0.47% and 0.4% of the respondents have chosen the opt-out options for rams and ewes, respectively. Such a small proportion of the opt-out options may indicate that the available alternatives are plausible. Given the very small choices, the opt-out options were ignored during data analysis.

Choice modeling and statistical analysis

We started the empirical analysis of preference data using a standard multinomial logit model. Estimation of utility coefficients using multinomial logit model requires the assumption of independence of irrelevant alternatives (IIA). However, the data could not satisfy the IIA assumption when tested using the Hausman-McFadden test. Fortunately, the generalized multinomial logit (G-MNL) model, derived from mixed and scaled multinomial logit models, does not require the assumption of IIA [23, 24]. Furthermore, the G-MNL model accounts for both taste and scale heterogeneity implying that utility estimates will not be affected by variations in the unobserved component of the model [24]. Mathematically, attribute utility using G-MNL model can be computed as:

$$U_{int} = [\sigma_n \beta + \gamma \eta_n + (1-\gamma)\sigma_n \eta_n] X_{int} + \varepsilon_{int} \quad (2)$$

Where U_{int} is the utility associated with the ith alternative chosen by the nth respondent (n = 1, …, 370) at t choice scenario (t= 1 to 4)), X_{int} is a vector of observed attribute levels of ram and ewe, β is a vector of mean attribute utility weight, η is s a random term associated with a person-specific deviation from the mean utility, ε_{int} is the idiosyncratic error term, σ_n represents the scale of the error term, γ is a parameter (value between 0 and 1).

Following equation 2, let *Ynit* = 1 if the respondent n has chosen the ith alternative at t choice situation, or 0 otherwise, the simulated probability choice in the G-MNL model takes the following form.

$$\hat{P}_n = \frac{1}{D}\sum_{d=1}^{D}\prod_t\prod_i\left(\frac{\exp(\sigma^d\beta+\gamma\eta^d+(1-\gamma)\sigma^d\eta^d)X_{nit}}{\sum_{k=1}^{i}\exp(\sigma^d\beta+\gamma\eta^d+(1-\gamma)\sigma^d\eta^d)X_{nkt}}\right)^{Y_{nit}} \quad (3)$$

Where $\sigma^d = \exp(\bar{\sigma} + \tau\varepsilon_0^d)$, η^d is a K-vector distributed with multivariate normal (0, $\sum$), τ is the standard deviation of the scale of the error term, whereas ε_0^d is a scalar with normal distribution *N*(0, 1). The simulation process requires d = 1… D draws for $\{\eta^d\}$ and $\{\varepsilon_0^d\}$.

Fiebig et al. [24] proposed different ways of implementing the G-MNL model by imposing restrictions on parameters. In the present study, we set the value γ to zero ($\gamma = 0$) to compute the utility coefficient. Given the difficulty of setting appropriate initial value, most researchers restrict the value of γ to zero.

Maximum simulated likelihood estimates, the goodness of fit of the model and the odds ratio were estimated using the function *mlogit* and *gmnl* packages of R software version 3.6.1 [25]. Data for ram and ewe trait preferences were analyzed separately. Socioeconomic characteristics of the respondents, such as sex, educational status, location, sheep flock size, and crop and grazing land sizes, were evaluated for possible influences on preferences of smallholder farmers. However, none of them has exerted a significant effect on trait preferences, thus omitted from G-MNL to keep models parsimony. Indices for ranking of sheep production objectives and ram and ewe selection criteria of the smallholder farmers were calculated for the first four ranks following König et al. [15] and Bett et al. [26].

$$Index = \sum_{n=1}^{4} a_m X_{nm} / \left(\sum_m \sum_{n=1}^{4} a_m X_{nm}\right) \quad (4)$$

Where a_m is the rank weight associated with trait or criteria m (a_1= 4, a_2= 3, a_3= 2, a_4=1), X_{nm} is the proportion of smallholder farmers who ranked the mth trait or criteria in the nth rank (n = 1 to 4 ranks), *m* represents sheep traits and the different purposes of sheep production.

Results

General characteristics of the respondents

From a total of 370 respondents, about 95.4% were male ~~while the remaining were female farmers~~. Such large variation was observed because the number of female-headed households who owned sheep and available for sampling during the study period was very small. All the respondents stated that their main occupation is agriculture, involving both crop and livestock productions. The mean crop and private grazing landholdings were 0.8 and 0.28 hectares, respectively. With respect to the literacy status of the respondents, 32.7% had attended informal and spiritual education systems and were able to read and write. Likewise, about 29.2% of the respondents had attended primary school while only 11.6% reached a post-primary school level. The remaining 26.5% of the respondents were illiterate.

Sheep production objectives

The importance of sheep production for the smallholder farmers was examined based on the overall index calculated from the proportions of four ranks (Table 2). Given the highest index value of 0.36, income generation was ranked as ~~a~~ the primary reason for keeping sheep. In addition, meat for consumption purpose and manure as organic fertilizer and local fuel sources were ranked as second and third objectives, respectively. Wool production was the least ranked objective of sheep production. However, nearly all respondents practiced the shearing of sheep at least once per year as part of controlling external parasite and have been ~~using~~ used the wool for traditional purposes.

Table 2. Rank proportions and index values for sheep production objectives at smallholder level.

Sheep production objectives	Rank proportions*				Index
	1	2	3	4	
**Income generation**	0.65	0.33	0.01	0.01	0.36
**Meat source**	0.32	0.52	0.14	0.03	0.31
**Saving/asset**	0.02	0.08	0.18	0.09	0.08
**Manure source**	0.00	0.07	0.45	0.44	0.16
**Sheep skin source**	0.00	0.01	0.22	0.42	0.09
**Wool production**	0.00	0.00	0.01	0.03	0.00

**1-4 represent rank 1, rank 2, rank 3 and rank 4*

Sheep flock size and composition

Mean flock size and standard deviations for each category of sheep and sampling districts are given in Table 3. The overall mean for sheep flock size of the study areas was 10.21, with a flock size range of 2 to 43 sheep. Across the study districts, sheep flock size was significantly larger in Lay Gayient than the two other districts (P<0.001). With the overall mean of 5.21 ewes, the proportion of breeding ewes is accounted for about 51% of the total flock size. Moreover, a significant difference exists among the three districts in the mean number of breeding ewes (P<0.001). Given the overall mean of 0.48, the number of breeding rams was generally small across the study districts. The ratio of breeding ram to ewes was 1:11. Within the flock, ewe and ram lambs represent those with age group between six months and one year, which can be used as a replacement for breeding flock or sold for income generation or otherwise slaughtered for consumption. Lambs represent both male and female groups with age below six months and account for about 26.6% of the total sheep flock.

Table 3. Sheep flock size and composition at smallholder level by the study districts.

Sheep category	Districts						Overall (N=370)*	
	Estie (N=117)		Farta (N=125)		Lay Gayient (N=128)			
	Mean	Std.	Mean	Std.#	Mean	Std.	Mean	Std.
**Breeding ewes**	5.15a	1.34	3.86b	1.19	6.64c	3.2	5.23	2.43
**Breeding rams**	0.52a	0.57	0.46a	0.50	0.45a	0.60	0.48	0.56
**Ewe lambs**	0.45a	1.03	0.64a	0.87	1.87b	1.53	1.01	1.34
**Ram lambs**	0.62a	0.94	0.79a	0.92	0.68a	1.15	0.70	1.01
**Lambs**	2.83a	1.33	2.76a	1.75	2.58a	2.16	2.72	1.79

**Castrated**	0.07a	0.31	0.03a	0.18	0.14a	0.68	0.08	0.45
**Total flock size**	9.65a	2.76	8.54a	3.40	12.36b	6.39	10.21	4.79

**N is the number of respondents, #Std. is the standard deviation, means in a row with different letters are significantly different (P<0.001)*

Ram use practices and mating management

Across the study districts, about 46.2% of the smallholder farmers have their own breeding rams with sources born in the flock (36.8%), purchased with partners (7.8%) and bought privately (1.6%). On the other hand, more than half (53.8%) of the respondents were without breeding rams but all had access for ram services from their neighbors and relatives. According to the view of the respondents, the average duration in which a particular ram staying within the flock for breeding purposes is about 2.26 years. All the respondents stated that mating is uncontrolled with year-round lambing. Given the absence of pedigree records coupled with the practices of using rams born within the flock, the level of inbreeding within the flock is expected to be high. While discussing with smallholder farmers, their understanding of inbreeding appeared to be minimal.

Ram and ewe selection criteria

About 91% of the respondents have been practicing selection of breeding rams and ewes within their own flock using different criteria. Index values calculated for smallholder farmers' selection criteria for breeding rams and ewes are given in Table 4. Given the highest index value of 0.34, body size was ranked as the primary selection criteria for breeding rams followed by coat color and tail type. Similarly, body size (index = 0.34) and coat color (index = 0.25) were two of the most important breeding ewe selection criteria. However, records about sheep performance and pedigree were nonexistent in all respondents involved in the study, thus selection decisions are mainly based on physically observable attributes of sheep. Given the very low index values, adaptation and wool yield traits were the least considered selection criteria for both sexes. Across

the study districts, there was no disparity in the order of major selection criteria for both rams and ewes.

Table 4. Rank proportions and index values for breeding ram and ewe selection criteria

**Selection criteria**	**Rank proportions and index (rams)***					**Rank proportions and index (ewes)**				
	1	2	3	4	Index	1	2	3	4	Index
**Body size**	0.64	0.21	0.09	0.02	0.34	0.63	0.26	0.05	0.02	0.34
**Litter size**	-	-	-	-	-	0.02	0.02	0.09	0.12	0.04
**Lamb survival**	-	-	-	-	-	0.00	0.00	0.01	0.05	0.01
**Lamb growth**	-	-	-	-	-	0.02	0.04	0.03	0.08	0.04
**Lambing interval**	-	-	-	-	-	0.02	0.05	0.13	0.21	0.07
**Ear size**	0.03	0.05	0.13	0.19	0.07	0.02	0.04	0.10	0.07	0.05
**Pedigree**	0.03	0.01	0.05	0.08	0.03	0.05	0.01	0.03	0.05	0.03
**Coat color**	0.25	0.44	0.16	0.08	0.27	0.22	0.41	0.15	0.06	0.25
**Growth rate**	0.01	0.04	0.07	0.08	0.04	0.01	0.04	0.08	0.05	0.04
**Sexual maturity**	0.00	0.02	0.02	0.10	0.02	0.00	0.06	0.19	0.12	0.07
**Libido**	0.01	0.03	0.10	0.18	0.05	-	-	-	-	-
**Tail type**	0.03	0.14	0.18	0.17	0.11	0.01	0.06	0.08	0.11	0.05
**Adaptation**	0.00	0.00	0.01	0.01	0.00	0.00	0.00	0.02	0.02	0.01
**Wool yield**	0.00	0.01	0.02	0.01	0.01	0.00	0.01	0.02	0.02	0.01
**Horn status**	0.00	0.02	0.02	0.10	0.06	0.00	0.00	0.01	0.00	0.00

**1-4 represent rank 1, rank 2, rank 3 and rank 4*

Culling criteria for rams and ewes

Nearly all smallholder farmers (97%) responded that both rams and ewes with small body sizes are not preferred for breeding purposes, thus excluded from the flock. Likewise, sheep with black coat color were not favored for breeding purposes in both sexes, given 94% of the smallholder farmers practiced culling due to unwanted coat color. Furthermore, the majority of the respondents said that rams and ewes could be culled due to old age and fertility problems. Based on the view of the respondent, the average culling age for breeding ewes due to oldness was 9.78 years while rams were excluded from the flock much earlier than becoming old. Moreover, poor mothering ability of ewes implicated largely on lambs' performance was reported to be one of the main culling reasons for female sheep. Conversely, the majority of the respondents did not cull rams

and ewes due to poor body condition. The respondents highlighted that body condition fluctuation due to seasonal variations in the availability of feed is very common in the study areas.

Choice preference for ram and ewe attributes

Utility coefficients estimated for ram and ewe attributes are presented in Table 5. Pseudo R-square (ρ^2) estimates of the model were 0.2 and 0.27 for ram and ewes, respectively, implying that the overall fit of the model is good. According to McFadden [27], ρ^2 values ranging from 0.2 to 0.4 are indicators for the excellent fit of a model. For each attribute, one of the levels was used as a reference with a value set to zero. Furthermore, body size and tail type for rams, and body size, growth rate and lambing intervals for ewes were fitted as random parameters. The selection of random parameters was based on the overall contribution to model fitness [23].

All attributes of breeding rams and ewes, included in the model as fixed and random parameters (Table 5), have shown significant influences on smallholder farmers' preference ($P<0.05$). In other words, smallholder farmers preferred a ram with good tail type, large body size, predominantly brown coat color and rapid growth in the presence of horn. However, large ear size with an estimated coefficient of -0.1869 was not preferred over ram with small ears. The magnitude of the utility coefficient shows that the tail type of ram is the most preferred attribute while ear size is least preferred. Regarding the choices for breeding ewes, all attributes resulted in positive utility coefficients as expected. Based on the magnitude of utility coefficients, good mothering ability was the most preferred ewe attributes followed by predominantly brown coat color and large body size. Likewise, short lambing interval and rapid growth rate were the fourth and fifth preferred attributes while good tail type and twining were placed in the bottom as least preferred ewe attributes.

Estimated coefficients for the standard deviation of random parameters for both rams and ewes were not significant (P>0.05), indicating that significant preference heterogeneity was not observed among respondents (Table 5). In contrast to the choice situations for ram attributes, the standard deviation of the scale parameter ($\tau = 0.7535$) revealed significant scale heterogeneity for ewe attribute choice scenarios (P<0.001).

Table 5. Estimates of smallholder farmers' preferences for breeding ram and ewe attributes.

	**G-MNL model (ram)**			**G-MNL model (ewe)**		
	Estimates	SE	P-value	Estimates	SE	P-value
**Fixed parameters**						
**1: Intercept***	0.0	-	-	0.0	-	-
**2: Intercept**	0.2863	0.117	0.014	1.1342	.251	<0.001
**3: Intercept**	-0.9403	0.163	<0.001	0.6060	0.236	0.01
**4: Intercept**	0.9929	0.152	<0.001	1.0351	0.241	<0.001
**Coat color (brown)**	0.3237	0.039	<0.001	1.1517	0.312	<0.001
**Growth rate (rapid)**	0.2666	0.043	<0.001	-	-	-
**Ear size (large)**	-0.1869	0.060	0.002	-	-	-
**Horn (horned)**	0.2523	0.041	<0.001	-	-	-
**Mothering ability (good)**	-	-	-	1.3056	0.357	<0.001
**Litter size (twin)**	-	-	-	0.7737	0.327	0.018
**Tail type (good)**	-	-	-	0.8547	0.240	<0.001
Random parameters						
**Body size (large)**	0.5132	0.048	<0.001	1.1287	0.333	<0.001
**Tail type (good)**	0.8986	0.051	<0.001	-	-	-
**Growth rate (rapid)**	-	-	-	0.9618	0.254	<0.001
**lambing interval (short)**	-	-	-	0.9645	0.23	<0.001
**The standard deviation of random parameters**						
**Body size (large)**	0.001	0.072	0.99	0.0055	0.07	0.94
**Tail type (good)**	0.014	0.318	0.96	-	-	-
**Growth rate (rapid)**	-	-	-	0.2086	0.244	0.39
**lambing interval (short)**	-	-	-	0.0745	0.171	0.66
**Tau (τ)**	0.004	0.13	0.96	0.7565	0.172	<0.001
**Gamma (γ)**	0	-	fixed	0	-	fixed
**Log likelihood null**	-1864.1	-	-	-1894.4	-	-
**Log likelihood function**	-1493.1	-	-	-1376.3	-	-
**Number of observations**	1473			1474	-	-
**McFadden's ρ^2**	0.20	-	-	0.27	-	-

**the first choice alternative was used as a reference category hence intercept is zero,*

The odds ratio for choosing ram and ewe attribute levels

~~The odds ratio of choosing ram and ewe attribute levels based on the G-MNL model are given in Table 6.~~ Accordingly, the odds of selecting a ram with good tail type is 2.46 times higher than a ram with bad tail type. (Table6) Similarly, a ram with large body size is 1.67 times more likely to be chosen by smallholder farmers than a ram with small body size. However, the choice of a ram having large ear size is less likely than a ram with small ear size. Concerning the odds of ewe attribute levels, ewe with good mothering ability is 3.69 times more likely to be picked up by smallholder farmers than ewe with poor mothering ability. Overall, all the preferred attribute levels of ewes have shown high odds ratios compared with the reference level of the corresponding attributes.

Table 6. The odds ratios for all levels of the different ram and ewe attributes.

	G-MNL model	
**Attributes**	Ram odd ratio (95% CI)*	Ewe odd ratio (95% CI)
**Body size (large vs small)**	1.67 (1.52 to 1.84)	3.09 (1.61 to 5.94)
**Coat color (brown vs white)**	1.38 (1.28 to 1.49)	3.16 (1.72 to 5.83)
**Growth rate (rapid vs slow)**	1.31 (1.20 to 1.42)	2.62 (1.59 to 4.31)
**Tail type (good vs bad)**	2.46 (2.22 to 2.71)	2.35 (1.47 to 3.77)
**Ear size (large vs small)**	0.83 (.74 to .93)	-
**Horn status (horned vs polled)**	1.29 (1.19 to 1.40)	-
**Lambing interval (short vs long)**	-	2.62 (1.67 to 4.12)
**Mothering ability (good vs poor)**	-	3.69(1.83 to 7.42)
**Twining (twin vs single)**	-	2.17 (1.14 to 4.12)

**95% lower and upper confidence intervals in the parenthesis*

Discussion

A good understanding of livestock production and the breeding system is fundamental to design a breeding program at the smallholder level [28, 29]. More importantly, for breeding programs to be operational at smallholder levels, the active involvement of smallholder farmers from planning to implementation is strongly advised by multiple scholars [13, 15, 30]. In this study, sheep breeding practices and trait preferences of smallholder farmers were identified using participatory

approaches. The results of the present study can be used to design village-based breeding programs for indigenous sheep inhabiting the northwest highlands of Ethiopia.

Sheep production objectives and flock structure

In the study areas, income generation and meat consumption were the main objectives of keeping sheep by smallholder farmers. Such objectives imply that indigenous sheep can play huge roles in poverty reduction at the smallholder level if adequate efforts are made to improve their productivity. Similar sheep production objectives have been reported in different parts of Ethiopia, particularly in the crop-livestock mixed farming system [4, 5, 14, 31].

The present study revealed that the average flock size per smallholder farmer is generally small. In-country wise, sheep flock size per household is reported to vary across production systems depending on the availability of inputs and dependence on livestock. For instance, Edea et al. [14] reported sheep flock size of 8 to 11 in crop-livestock mixed production system while Nigussie et al. [32] reported an average flock size of about 97 and 72 sheep in Pastoral and agro-pastoral production systems, respectively. For breeding programs targeting genetic improvement through selection, the small number of sheep available at the individual farmer level could be problematic, for instance, from the perspectives of minimizing inbreeding and obtaining optimal genetic gain. In such scenarios, establishing strong collaboration among smallholder farmers to create large breeding flock is fundamental for the sustainable use of genetic resources.

Sheep breeding practices

Valuing indigenous knowledge is vital to ensure the sustainability of a breeding program intended to be implemented at the smallholder level [29, 30]. Interestingly, the majority of the smallholder farmers in the study areas have been practicing selection within their own sheep flock using diverse criteria. It was found that body size and coat color were two of the most important selection criteria

for both breeding rams and ewes. Biologically, coat color has a qualitative nature implying that it cannot be measured on a scale basis. Large body size, on the other hand, can be expressed in terms of linear body measurements such as body length, height, pelvic and girth circumferences. In addition, linearly measured body size traits are easy to measure even at smallholder level and are reported to be moderately heritable [33]. Such facts imply that smallholder farmers could indeed achieve some level of improvement when applying selection based on body size.

Furthermore, although the growth rate was not ranked among the top selection criteria in both sexes, it has been known to have moderate to high genetic and phenotypic correlations with body size traits that can be measured linearly [33, 34]. Thus, body size based selection practices of the smallholder farmers could have a positive impact on the growth rate. Next to body size and coat color, tail type and ear size were the third and fourth selection criteria for breeding rams. Naturally, sheep breeds in the study areas are generally characterized by fatty tail type, horizontally oriented ear and males are often horned [2, 35]. For breeding ewes, female reproductive traits such as lambing interval, age at first lambing and litter size were the selection criteria considered following body size and coat color.

Another important experience of smallholder farmers in the study areas is the practice of culling of sheep perceived as not suitable for breeding purposes within their own flock. Reasons for culling were due to small body size, unfavorable coat color, old age and fertility problems for both male and female sheep, and due to poor mothering ability of ewes. Such practices are also reported to be implemented by smallholder farmers in other parts of Ethiopia [36]. In the study areas, the average production lifetime of breeding ewes is about 9.55 years implying that smallholder farmers often keep preferred ewes for a longer period. Breeding rams, on the other hand, stay within the flock relatively for short period, which is around 2.26 years on average

indicating that rams provide breeding services for about one year considering a year of sexual maturity age.

The practice of using rams for short duration is assumed to be useful to minimize inbreeding within the flock. Yet, about 36.8% of the smallholder farmers used rams born in the flock, which could result in mating between genetically related sheep thereby increase inbreeding. The issue of inbreeding at smallholder levels has been also indicated in other parts of Ethiopia [4, 14, 36] and elsewhere in West Africa [37]. Although pedigree-based records are lacking, the practice of random mating and the use of communal rams can be considered as encouraging breeding practices to reduce the effect of inbreeding in the study areas.

Choice preference for sheep traits

Choice modeling was applied to elucidate the preference of smallholder farmers for indigenous sheep traits. Among ram attributes, a good tail type was the most preferred attribute followed by large body size and predominantly brown coat color. These attributes were also among the major ram selection criteria although the order of importance is reversed. The choice of ram attributes seemed to be a reflection of the existing traditional breeding approach implemented in the study areas. Duguma et al. [10] performed a choice modeling in four Ethiopian indigenous sheep breeds (Horro, Menz, Afar and Bonga). These authors reported that sexual activity (libido) for Horro and Menz, good tail type for Bonga and color type for Afar sheep breeds were the most preferred ram attributes. Although libido attribute was not included in the choice experiment of the present study, it was ranked as the fifth ram selection criteria by the smallholder farmers (Table 4). Such preference heterogeneity for ram attributes could likely be due to differences in sheep breed, production system and sociocultural characteristics of smallholder farmers.

Rapid growth rate and the presence of horns were also found to be important ram attributes given their significant influence on the preference of smallholder farmers. Growth related traits, such as weight at different ages, are easy to measure and have reasonably high heritability. Due to such characteristics, growth traits have been the main targets of breeding programs piloted for a few indigenous sheep breeds in Ethiopia [38, 39]. Furthermore, the earlier attempts of selection for body weight on nucleus herds have shown that Ethiopian indigenous sheep are reasonably responsive for selective breeding [40]. Unexpectedly, a ram with small ear size was preferred than large size although ear size is the least important attribute in terms of the magnitude of utility coefficient. Based on the index value in Table 4, ear size was ranked as the fourth ram selection criteria implying that the emphasis of smallholder farmers toward ear size is not strong.

Regarding the preference for ewe attributes, good mothering ability, representing the nourishing potential of ewes for better growth and survival of lambs, was the most preferred attribute. This result is in agreement with Duguma et al. [10] who reported a high choice preference for good mothering ability of ewes in four indigenous sheep breeds of Ethiopia. The strong preference for mothering quality could be a good indicator of the mechanism by which smallholder farmers are trying to be profitable even under low input sheep production system. One evidence could be is that smallholder farmers often sell lambs for income generation, thus well-nourished lambs are expected to fetch a better price.

Besides good mothering ability, predominantly brown coat color, large body size, short lambing interval, and rapid growth rate were the preferred attributes of breeding ewes by smallholder farmers. Although the tail type and litter size strongly influenced the preference of smallholder farmers, both were less important compared to other ewe attributes. This could indicate that the main emphasis of smallholder farmers for ewe choice is lamb production while

focused largely on observable characteristics for ram profiles. However, for ewe attributes other than good mothering ability, smallholder farmers' preference obtained in the present study was not in agreement with the findings of Duguma et al. [10]. This clearly highlights the importance of evaluating sheep trait preferences on a breed basis prior to designing a breeding program.

One appealing feature of choice modeling using G-MNL is the possibility of accommodating choice heterogeneity among respondents. Unlike breeding ram, ewe preference analysis had revealed significant scale heterogeneity ($P<0.001$). However, taste heterogeneity in both sexes was not statistically significant. Scale heterogeneity could occur due to variations in the choice behavior of respondents [24]. For instance, for some respondents, the choice may be driven by one or few attributes resulting in a very small estimate for the scale of the error term or a very large utility coefficient. Such choice characteristics could likely be the source of scale heterogeneity in the present study. Fortunately, the G-MNL model takes into account both taste and scale heterogeneity simultaneously. Thus, maximum simulated likelihood estimates of parameters will not be biased due to heterogeneity.

Potential traits for breeding objective

The present study revealed that smallholder farmers have given due emphasis for both quantitative and qualitative attributes of indigenous sheep. Given the easiness of measurement and heritable nature of the traits, body size and growth rate can be the main components of sheep breeding objective in the study areas. Linear body measurements such as body length, chest girth, pelvic width and height at wither are proxy traits for body size while growth can effectively be described in terms of weight at different ages. Furthermore, lambing interval, early growth and survival of lambs should be incorporated in the breeding objective. Although the latter two traits were not

among the main ewe selection criteria, they are more likely to reflect the mothering ability of ewe that highly influenced smallholder farmers' preference.

However, qualitative traits such as tail type and coat color cannot be measured on a scale basis, thus difficult to incorporate directly in the breeding objective despite their significant effects on preference. This implies selection decision at smallholder level should not only be based on estimated breeding value of traits in the breeding objective but instead, additional preferences of farmers should be taken into accoun[illegible]ecause it has been said that more than any form of financial support, satisfying the interest of smallholder farmers is vital for the sustainability of genetic improvement programs [30]. Overall, production, reproduction and adaptive traits could be combined alternatively for optimal genetic gain without making the breeding objective more complex.

Conclusion

Although average flock size per head is small, the majority of the smallholder farmers have experiences in selecting and culling of rams and ewes within their own sheep flock. The absence of pedigree records and the use of rams born in the flock could increase the level of inbreeding. Based on the magnitude of utility coefficients, a good tail type was the most preferred ram attribute followed by large body size, predominantly brown coat color and rapid growth rate. Similarly, smallholder farmers have shown their highest preference for good mothering ability of ewes followed by predominantly brown coat color, large body size, short lambing interval, and rapid growth rate. The present results implied that breeding objectives incorporating production, reproduction and adaptive sheep traits can be derived by alternatively combining highly preferred attributes having measurable and heritable characteristics. However, selection decisions at the

smallholder level should not only be based on the outcome of traits included in the breeding objective but instead, additional preferences of farmers need to be taken into account.

Acknowledgments

The corresponding author would like to thank smallholder farmers from Estie, Farta and Lay Gayient districts who spent their invaluable time to provide the required data for this study.

References

1. Central Statistical Agency of Ethiopia (CSA). Agricultural Sample Survey 2017/18. Volume II report on livestock and livestock characteristics (private peasant holdings). Statistical bulletin 587. 2018 April [cited 2019 August 20]. Available at: http://www.csa.gov.et/survey-report/category/370-eth-agss-2018.
2. Gizaw S, Komen H, Hanotte O, Van Arendonk JAM. Indigenous sheep resources of Ethiopia: types, production systems and farmers preferences. Animal Genetic Resources Information, 2008; 43:25–39.
3. Mekuriaw S, Mekuriaw Z, Taye M, Yitayew A, Assefa H, Haile A. Traditional management system and farmers' perception on local sheep breeds (Washera and Farta) and their crosses in Amhara Region, Ethiopia. Livestock Research for Rural Development, 2012; 24(1). Available at: http://www.lrrd.org/lrrd24/1/meku24004.htm.
4. Getachew T, Haile A, Tibbo M, Sharma AK, Sölkner J, Wurzinger M. Herd management and breeding practices of sheep owners in a mixed crop-livestock and a pastoral system of Ethiopia. African Journal of Agricultural Research, 2010; 5(8):685-691.

5. Dagnew Y, Urge M, Tadesse Y, Gizaw S. Sheep production and breeding systems in northwestern lowlands of Amhara Region, Ethiopia: Implication for conservation and improvement of Gumz sheep breed. Open Journal of Animal Sciences, 2017; 7:179-197.
6. Mekuriaw S, Mekuriaw Z, Taye M, Mekuriaw G, Amane A, Bimrew, et al. Growth performance and linear body measurements of Washera, Farta and their crossbreed sheep under farmers management system in western highland of Amhara Region. Scientific Journal of Veterinary Advances, 2013; 2(9):132-143.
7. Gulilat L, Walelign E, Amane A. Evaluation of the effects of concentrate supplementation on carcass merits of Farta sheep. Academic Research Journal of Agricultural Science Research, 2018; 6(1): 35-41.
8. Gizaw S, Van Arendonk JAM, Komen H, Windig JJ, Hanotte O. Population structure, genetic variation and morphological diversity in indigenous sheep of Ethiopia. Animal genetics, 2007; 38:621-628.
9. Bett HK, Bett RC, Peters KJ, Kahi AK, Bokelmann W. Estimating farmers' preferences in selection of indigenous chicken genetic resources using non-market attributes. Animal Genetic Resources, 2011; 49:51–63.
10. Duguma G, Mirkena T, Haile A, Okeyo AM, Tibbo M, Rischkowsky B, et al. Identification of smallholder farmers and pastoralists' preferences for sheep breeding traits: a choice model approach. Animal, 2011; 5(12):1984–1992.
11. Woldu T, Markemann A, Reiber C, Kassie GT, Zárate AV. Combining revealed and stated preferences to define goat breeding objectives in Ethiopia. Livestock Science, 2016; 191:179–186.

12. Terfa ZG, Garikipati S, Kassie GT, Dessie T, Christley RM. Understanding farmers' preference for traits of chickens in rural Ethiopia. Agricultural Economics, 2019; 50:451–463.
13. Gizaw S, Komen H, van Arendonk JAM. Participatory definition of breeding objectives and selection indexes for sheep breeding in traditional systems. Livestock Science, 2010; 128:67–74.
14. Edea Z, Haile A, Tibbo M, Sharma AK, Sölkner J, Wurzinger M. Sheep production systems and breeding practices of smallholders in western and south-western Ethiopia: Implications for designing community-based breeding strategies. Livestock Research for Rural Development, 2012; 24 (7). Available at: http://www.lrrd.org/lrrd24/7/edea24117.htm.
15. König EZ, Mirkena T, Strandberg E, Audho J, Ojango J, Malmfors B, et al. Participatory definition of breeding objectives for sheep breeds under pastoral systems--the case of Red Maasai and Dorper sheep in Kenya. Tropical animal health and production, 2016; 48(1): 9–20.
16. Haile A, Wurzinger M, Mueller J, Mirkena T, Duguma G, Mwai O, et al. Guidelines for setting up community-based sheep breeding programs in Ethiopia. ICARDA tools and guidelines No.1, International Center for Agricultural Research in the Dry Areas; 2011[cited 2019 September 24]. Available at: https://cgspace.cgiar.org/bitstream/handle/10568/16486/GuidelinesSheepBreeding.pdf?sequence=6&isAllowed=y.
17. Duguma G, Mirkena, T. Haile, A. Iñiguez L, Okeyo AM, Tibbo M, et al. Participatory approaches to investigate breeding objectives of livestock keepers. Livestock Research for Rural Development, 2010; 22 (4). Available at: http://www.lrrd.org/lrrd22/4/dugu22064.htm.
18. Hensher DA, Rose JM, Greene WH. Applied choice analysis: A primer. Cambridge: Cambridge University Press; 2005.
19. SAS Institute Inc. New Features in JMP® 14. Cary, NC: SAS Institute Inc.; 2018.

20. Box GEP, Hunter JS, Hunter WG. Statistics for experimenters: design, innovation, and discovery. 2nd ed. Wiley, New Jersey; 2005.

21. Johnson R, Orme B. Getting the most from CBC. Sequim: Sawtooth Software Research Paper Series, Sawtooth Software. 2003 [cited 2019 January 10]. Available at: https://www.sawtoothsoftware.com/support/technical-papers/cbc-related-papers/getting-the-most-from-cbc-2003.

22. Orme BK. Getting Started with Conjoint Analysis Strategies for Product Design and Pricing Research. 2nd ed. Research publishers LLC; 2010.

23. Greene WH, Hensher DA. The Mixed Logit model: The state of practice. Transportation, 2003; 30: 133–176.

24. Fiebig DG, Keane MP, Louviere J, Wasi N. The Generalized Multinomial Logit Model: Accounting for Scale and Coefficient Heterogeneity. Marketing Science, 2010; 29 (3):393–421.

25. R Core Team. R: A Language and Environment for Statistical Computing. R Foundation for Statistical Computing, Vienna, Austria; 2019. Available at http://www.R-project.org.

26. Bett RC, Kosgey IS, Kahi AK, Peters KJ. Analysis of production objectives and breeding practices of dairy goats in Kenya. Tropical Animal Health and Production, 2009; 41: 307–320.

27. McFadden D. Quantitative methods for analyzing travel behavior of individuals: Some recent developments. Cowles Foundation discussion paper No. 474. 1977 November 22 [cited 2019 April 20]. Available at: https://cowles.yale.edu/sites/default/files/files/pub/d04/d0474.pdf.

28. Sölkner J, Nakimbugwe H, Zárate VA. Analysis of determinants for success and failure of village breeding programs: Proceedings of the 6th World Congress on Genetics applied to livestock production, 1998 January 11-16, Armidale, New England: University of New England; 1998.

29. Haile A, Mirkena T, Duguma G, Wurzinger M, Rischkowsky B, Tibbo M, et al. Community-based sheep breeding programs: Tapping into indigenous knowledge. Livestock Research for Rural Development, 2013; 25(12). Available at http://www.lrrd.org/lrrd25/12/hail25219.htm.

30. Wurzinger M, Sölknera J, Iniguez L. Important aspects and limitations in considering community-based breeding programs for low-input smallholder livestock systems. Small Ruminant Research, 2011; 98: 170–175.

31. Amare B, Kefyalew A, Zeleke M. Typical features, characterization and breeding objectives of Begait sheep in Ethiopia. Animal Genetic Resources, 2012; 51:117–123.

32. Nigussie H, Mekasha Y, Kebede K, Abegaz S, Pal SK. Indigenous sheep production system in eastern Ethiopia: Implications for genetic improvement and sustainable use. American Scientific Research Journal for Engineering, Technology, and Sciences, 2015; 11(1): 136-152.

33. Jafari S, Hashemi A. Estimation of genetic parameters for body measurements and their association with yearling live weight in the Makuie sheep breed. South African Journal of Animal Science, 2014; 44 (2):140-147.

34. Janssens S, Vandepitte W. Genetic parameters for body measurements and linear type traits in Belgian Bleu du Maine, Suffolk and Texel sheep. Small Ruminant Research, 2004; 54: 13–24.

35. Bimerow T, Yitayew A, Taye M, Mekuriaw S. Morphological characteristics of Farta sheep in Amhara Region, Ethiopia. Online Journal of Animal Feed Research, 2011; 1(6):299-305.

36. Gebreyowhens W, Tesfay Z, Tesfay Y. Management and breeding objectives of indigenous smallholder highland sheep in northern Ethiopia. Journal of Biology, Agriculture and Healthcare, 2016; 6 (1):96-106.

37. Jaitnera J, Soweb J, Secka-Njieb E, Demp L. Ownership pattern and management practices of small ruminants in The Gambia: Implications for a breeding program. Small Ruminant Research, 2001; 40:101-108.

38. Gizaw S, Komen H, van Arendonk JAM. Optimal village breeding schemes under smallholder sheep farming systems. Livestock Science, 2009; 124:82–88.

39. Mirkena T, Duguma G, Willam A, Wurzinger M, Haile A, Rischkowsky B, et al. Community based alternative breeding plans for indigenous sheep breeds in four agro-ecological zones of Ethiopia. Journal of Animal Breeding and Genetics, 2012; 129(3):244–253.

40. Gizaw S, Getachew T, Tibbo M, Haile A, Dessie T. Congruence between selection on breeding values and farmers' selection criteria in sheep breeding under conventional nucleus breeding schemes. Animal, 2011; 5(7): 995-1001.

Supporting information

S1 file. Raw data for respondents' general characteristics and sheep breeding practices.

It is an SPSS file with all the variables explained fully in the variable view section

S2 file. Raw data for breeding ram and ewe choice preferences.

It is an Excel file with all ram and ewe variables explained fully in the third cell of the sheet.

S3 text. R script for breeding ram and ewe choice preference data analysis.

R script for ram and ewe choice preferences are available separately but contained in the same file.

Click here to download Figure Fig1.tif

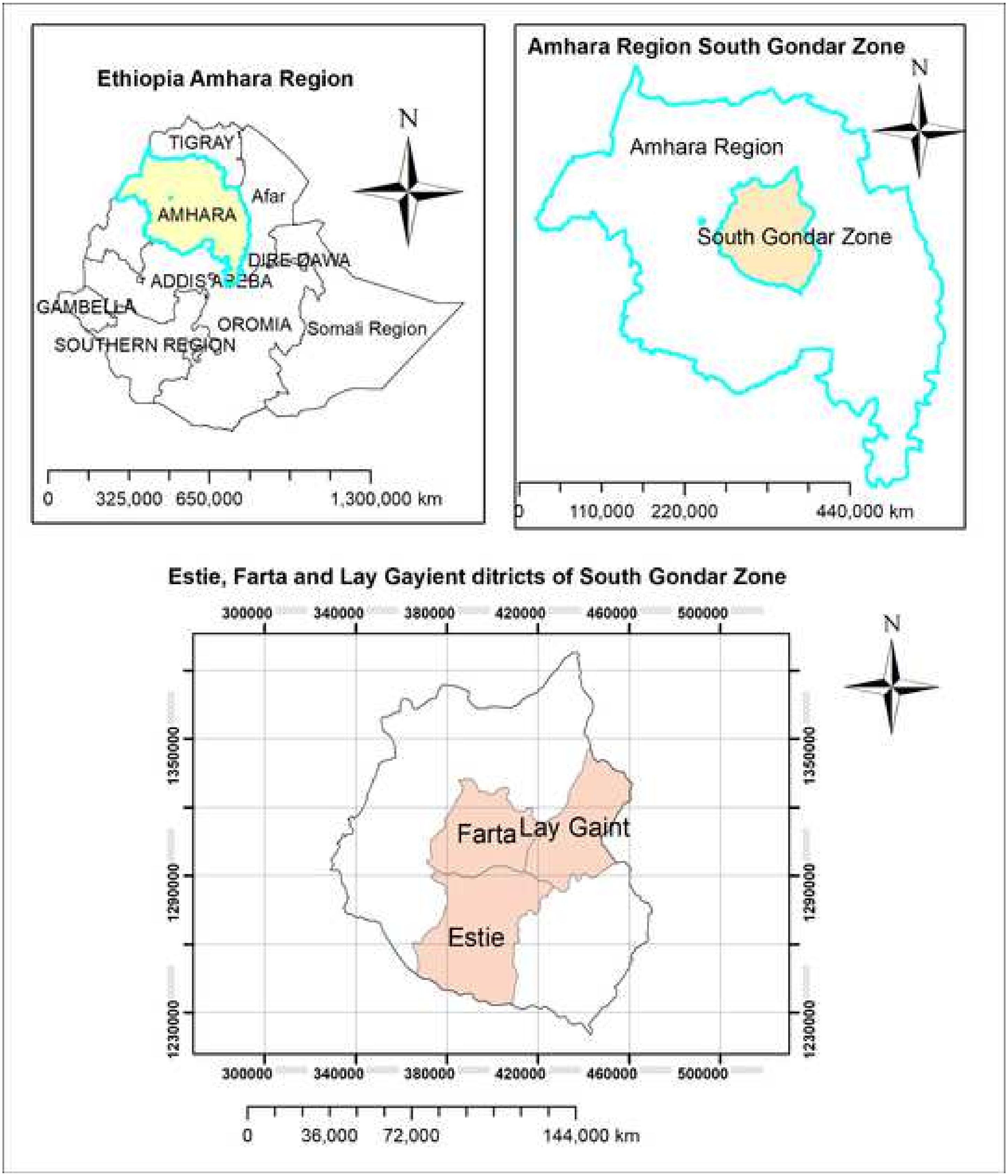

Figure

Click here to download Figure Fig2.tif

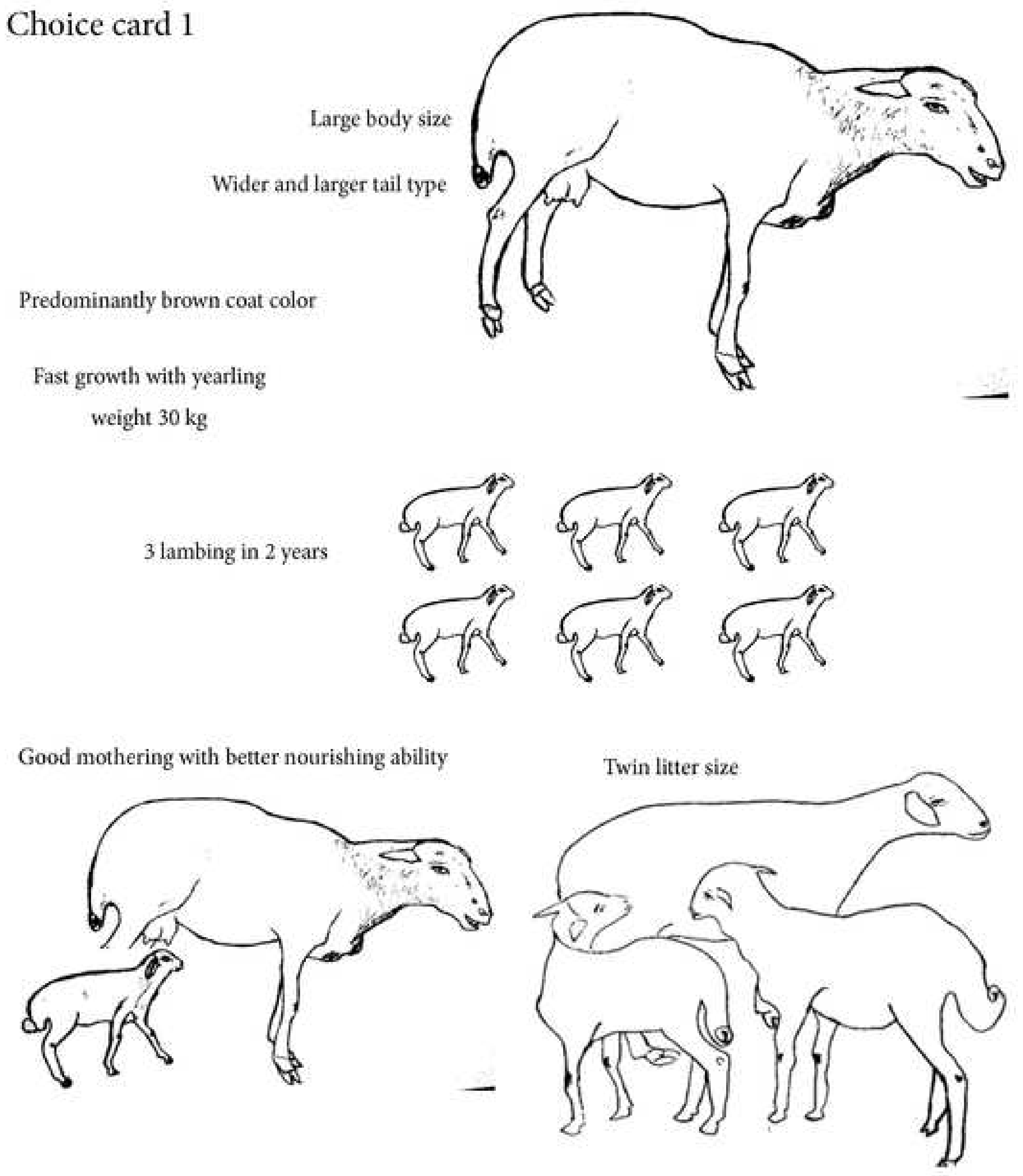

Click here to access/download
Supporting Information - Compressed/ZIP File Archive
supporting_information.zip
